# Automatic Rice Crop Height Measurement Using a Field Server and Digital Image Processing

**DOI:** 10.3390/s140100900

**Published:** 2014-01-07

**Authors:** Tanakorn Sritarapipat, Preesan Rakwatin, Teerasit Kasetkasem

**Affiliations:** 1 Geo-Informatics and Space Technology Development Agency (Public Organization), 120, The Government Complex (Building B), Chaeng Wattana Road, Laksi District, Bangkok 10210, Thailand; E-Mail: tanakorn.sri@gistda.or.th; 2 Faculty of Engineering, Kasetsart University, Jatujak, Bangkok 10900, Thailand; E-Mail: fengtsk@ku.ac.th

**Keywords:** rice crop height measurement, field server, digital image processing, image segmentation

## Abstract

Rice crop height is an important agronomic trait linked to plant type and yield potential. This research developed an automatic image processing technique to detect rice crop height based on images taken by a digital camera attached to a field server. The camera acquires rice paddy images daily at a consistent time of day. The images include the rice plants and a marker bar used to provide a height reference. The rice crop height can be indirectly measured from the images by measuring the height of the marker bar compared to the height of the initial marker bar. Four digital image processing steps are employed to automatically measure the rice crop height: band selection, filtering, thresholding, and height measurement. Band selection is used to remove redundant features. Filtering extracts significant features of the marker bar. The thresholding method is applied to separate objects and boundaries of the marker bar *versus* other areas. The marker bar is detected and compared with the initial marker bar to measure the rice crop height. Our experiment used a field server with a digital camera to continuously monitor a rice field located in Suphanburi Province, Thailand. The experimental results show that the proposed method measures rice crop height effectively, with no human intervention required.

## 1. Introduction

The demand for food is growing because of the continued increase in the World's population. Therefore, automated tools to improve agricultural productivity have received a great deal of attention recently. Automatic monitoring systems are one interesting approach to improve farming efficiency. The use of field servers or automatic weather stations has been suggested as a solution to continuously monitor the status of a crop [1–7]. Usually, a field server consists of various sensors, one or two cameras, and communication and control units. The devices can collect various types of data such as rain volume, humidity, wind speed, temperature, and soil moisture to support agricultural planning and management. The server can transfer data and be controlled remotely by a computer connected via cellular Internet or other wireless communication technologies.

A number of researchers have applied field servers known as smart monitoring systems for various applications. For example, Fukatsu *et al.* [8] developed a field server with a web server to monitor environmental parameters of interest via the Internet. Several studies [9,10] have tried to link field servers with a wired and wireless sensor network system that includes various meteorological sensors, cameras and a wireless LAN to provide information which can be accessed in web-based applications via a wireless communication system. Field servers are controlled by a management program called an agent system [11], which automatically collects sensor data at the field via Internet-based communication. The agent system can automatically operate the field sensor to follow user requirements, and can also perform a wide range of data analyses by using external processing techniques [12].

An application of field servers and their sensor technology for helping farm management in Taiwan was presented by Wan *et al.* [13]. This research used the ZigBee protocol to control remote sensors for poultry management. Wan *et al.* [13] also presented another use of sensor technology to achieve integrated automation management via the Internet for improving farm work. Similar work by Fukatsu [14] has developed a method to estimate occurrence of the insect pest *Leptocorisa chinensis* using synthetic attractants, a field server and image analysis.

The main goal of installing a field server in a farm is to monitor the plant growth so that resources such as fertilizer and water can be optimally utilized. There are many possible measures of plant growth including leaf area index, vegetation index, and plant height. Among them, the plant height is the most direct measurement of the plant growth and is directly related to productivity and growth rate. For example, under normal conditions, plants will grow to a certain height in each growth state. In contrast, if plant is distressed due to disease or a lack of water, its growth rate may drop, and consequently, the yield will be lower [15,16]. Several researchers, therefore, have investigated on how to measure plant height. In Qing [17], tree height measurement methods and tools have been introduced since tree height is one of the most important factors in forest resource management. These methods give excellent accuracy since tree height is directly measured by field surveys but these have high operational cost. To reduce the cost, the tree height measurement methods based on image processing techniques have been investigated by many researchers [18]. A tree height measurement method based on 3-D perspective view was presented by Zhang and Huang [19]. Han [20] developed a tree height measurement method based on 3D view with 3-point correction. These methods provide good accuracy at low cost because they do not require surveying. In addition, Zhang *et al.* [21] measured the height of *Miscanthus giganteus* grass based on Light Detection and Ranging (LIDAR). They used a SICK LMS 291 LIDAR to get data for crop height analysis. This sensor provides high accuracy but it is very expensive when compared with common digital cameras.

Some researchers have used tree height measurement approaches based on remote sensing technology. For example, the synthetic aperture radar (SAR) is a widely used sensor in measuring tree height [22–24]. Another technology that can provide a very accurate tree height measurement is the airborne LIDAR [25–31]. Unfortunately, these remote sensing methods have several disadvantages, including high cost and the difficulty of acquiring frequent real-time data, which may be essential for some analysis models that need a continuous data stream, such rice crop growth models, and for some applications that need real-time analysis such as rice disease monitoring. The work [32,33] by measured the height of rice crop as the distance between ground level to the tip of the panicle [32], and used this distance as one of the factors to indicate the yield potential [33–36].

In most literature, the height of rice crops is measured by a field survey. This approach produces very high precision crop height measurements, but is very costly [33,37]. Furthermore, field surveys can only be conducted a couple of times during one crop season. Hence, any sudden changes in crop status due to diseases or water shortage may be missed. As a result, we propose a new approach to continuously measure rice crop height using image processing techniques.

In order to acquire plant height information frequently at a low cost, this paper investigates an automatic method for rice crop height measurement using digital image processing techniques on a field server equipped with a digital camera. The idea is to set up a field server to take daily photos of a field where a marker bar of known height is also installed in the camera's field of view. As the rice grows higher, it obscures more parts of the marker bar. Thus, in the images, rice crop height can be indirectly measured by measuring the visible portion of the marker bar. To calculate the height, our proposed method uses digital image processing techniques consisting of band selection, filtering and thresholding. The benefits of our method are reduced manual work, lower costs, fast computation and real time availability of information.

This paper is organized as follows: Section 2 gives the details about: (1) study area; (2) the field server for monitoring rice crop field; and (3) our rice crop height measurement algorithm based on image processing techniques. Our experiments and the results are described in Section 3. Finally, Section 4 provides the conclusions of this paper.

## 2. Study Area, Field Server and Rice Crop Height Measurement Technique

### 2.1. Study Area

Our experimental area was a rice field located at 14.4742N (latitude in decimal degrees), 105.122E (longitude in decimal degrees) in Suphanburi, Thailand. Suphanburi is located in the central part of Thailand, about 150 km from Bangkok. The terrain of Suphanburi province is mostly low river plains, with small mountain ranges in the north and the west of the province. The southeastern part with the very low plain of the Tha Cheen River is a rice paddy area. This highly suitable area was selected to be our experimental area for rice crop monitoring. The field server was installed on 14 June 2012 for collecting various data needed for a rice crop analysis model.

### 2.2. Field Server

The field server is composed of sensors, cameras, communication units, control units and a power supply unit. The sensors are used to measure the characteristics of the environment. Our field server provides various sensor types consisting of temperature, atmospheric pressure, light, wind, and soil moisture. We installed two digital Single-Lens Reflex (SLR) cameras in the field server. The main function of the digital cameras is to convert an optical image into electronic signals. Communication units are used to transmit sensor and command data between the field server and a computer server. Control units are used to modify the actions of the field server. The power supply unit supplies electric power to electrical loads.

**Figure 1.**
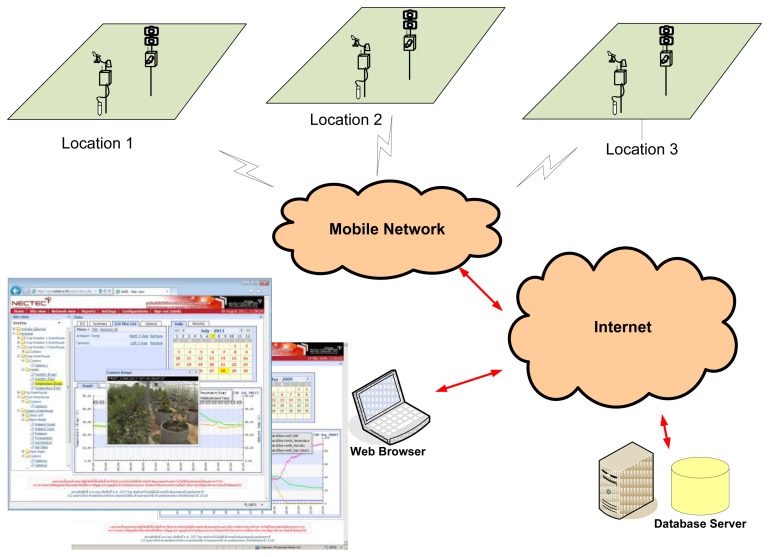
Field server system.

In our field server system, the control unit is programmed to manage the sensors and cameras included in the system. When a sensor measures some environmental properties, the observed data are stored in the unit and then transferred to a remote server. The collected data can then be viewed and managed through a web browser. The data are separated into two display types: (1) log data for common sensors; (2) image series for camera sensors. The architecture of the field server system is shown in Figure 1.

**Figure 2.**
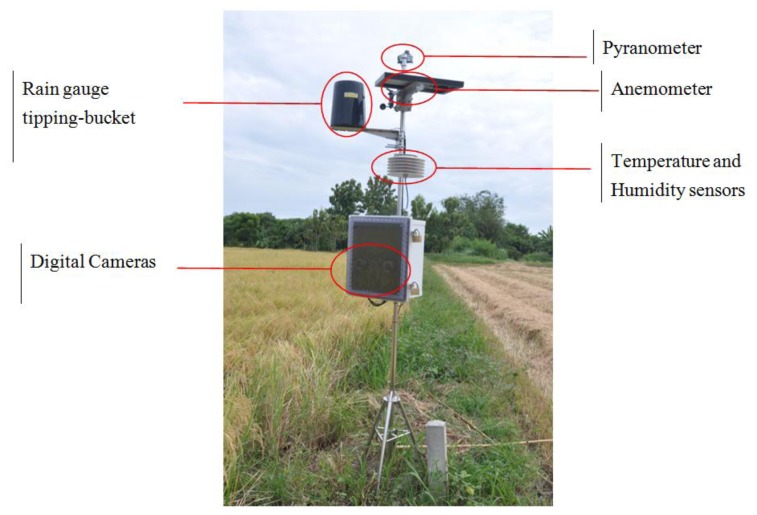
Field server in rice crop field in Suphanburi Province, Thailand.

Details of the sensors in the field server are as follows: a tipping-bucket rain gauge is an instrument to gather and measure the amount of liquid precipitation over a set period of time. An anemometer is a device for measuring wind speed, and is a common weather station instrument. Anemometers can be divided into two classes: those that measure the wind's speed, and those that measure the wind's pressure. A pyranometer is used to measure broadband solar irradiance on a planar surface; it is designed to measure the solar radiation flux density. A soil moisture sensor measures the amount of moisture found in the soil, which is important for agriculture. This sensor can measure moisture right near the roots. The most relevant parts to this research are the two Digital Single-Lens Reflex (DSLR) cameras with a mirror and prism system. One of these cameras provides RGB images for red, green and blue color bands. Figure 2 shows the field server in the rice field in Suphanburi Province, Thailand. Although the field server provides various sensors that give a wealth of data that can be useful for agricultural management, the main sensors used in the crop height measurement are one of the two DSLR cameras. Here, the DSLR camera used in the field server to acquire images in the rice crop field under natural conditions is a Canon 1100D (Canon Inc., Tokyo, Japan) with a cost of around $300. We decided to use DSLR cameras instead of a LIDAR sensor in measuring the crop height since the cost of LIDAR sensor can be as much as $6,000.

During image collection, the camera is always located above the scene and focused on the rice crop. We also fixed the location of the field server including the focus of the camera in this experiment. The digital images were stored as 24-bit color images with a resolution of 720 × 480 pixels and saved in RGB color space in the JPEG format. Although the camera can provide higher resolution images and using the higher resolution can give the higher accuracy, we did not use the higher or full resolution due to some limitations of other devices such as limited storage space, and data transfer rate of the communication module. As a result, we chose to use a lower resolution in order to reduce the amount of data storage space required, and enable more efficient data communication. This also indicates that our method can be used with other low-cost, low-resolution cameras. The resolution must be chosen such that the images are sharp enough for crop height estimation. The images are stored in the field server memory and then transferred to a server computer located at National Electronics and Computer Technology Center (NECTEC) for web service management, and then the images are transferred and stored at the Geo-Informatics and Space Technology Development Agency (GISTDA) for computing rice crop height. Then all the information of the field server including rice crop height is integrated to compute a rice crop model.

Rice crop height can be directly measured by field surveying. This technique provides the most accurate results, but is rather expensive in terms of high cost in human resources and time. Consequently, rice crop height can also be indirectly measured from a digital image by measuring the height of a marker bar installed in the field in the focus area of the digital camera. The marker bar is defined as a referenced height. In agricultural fields farmers commonly used a red-white stipe bar to estimate the height of rice plants since it is easy to evaluate the height for human evaluation by considering it block by block. In image analysis, using a uniform color bar makes it easier to detect than using a stripe bar. In this work, we still used the marker bar with alternating color instead of uniform color since we need to preserve the option of doing manual measurements giving reliable results and we did not want to make a new marker bar that requires more budget. In addition, image processing techniques can solve the problem of alternating colors effectively. In the rice crop field, the marker bar is 250 cm in height, with 25 alternating white and red stripes. Each stripe has a width of 10 cm. We put the marker bar into the soil at a depth of 20 cm (two stripes), and hence 230 cm (23 stripes) of the marker bar height was above ground level. A human can manually measure rice crop height by counting alternating color stripes and adding fractions of stripe. Rice crop height is the initial marker bar height minus the visible marker bar height. This is the logic behind our proposed method. However, we use digital image processing to measure the visible height of the marker bar and thus provide automatic rice crop height measurement, instead of requiring human evaluation. We used human evaluation as a standard to measure the accuracy of our automated technique.

### 2.3. Rice Crop Height Measurement Technique

**Figure 3.**
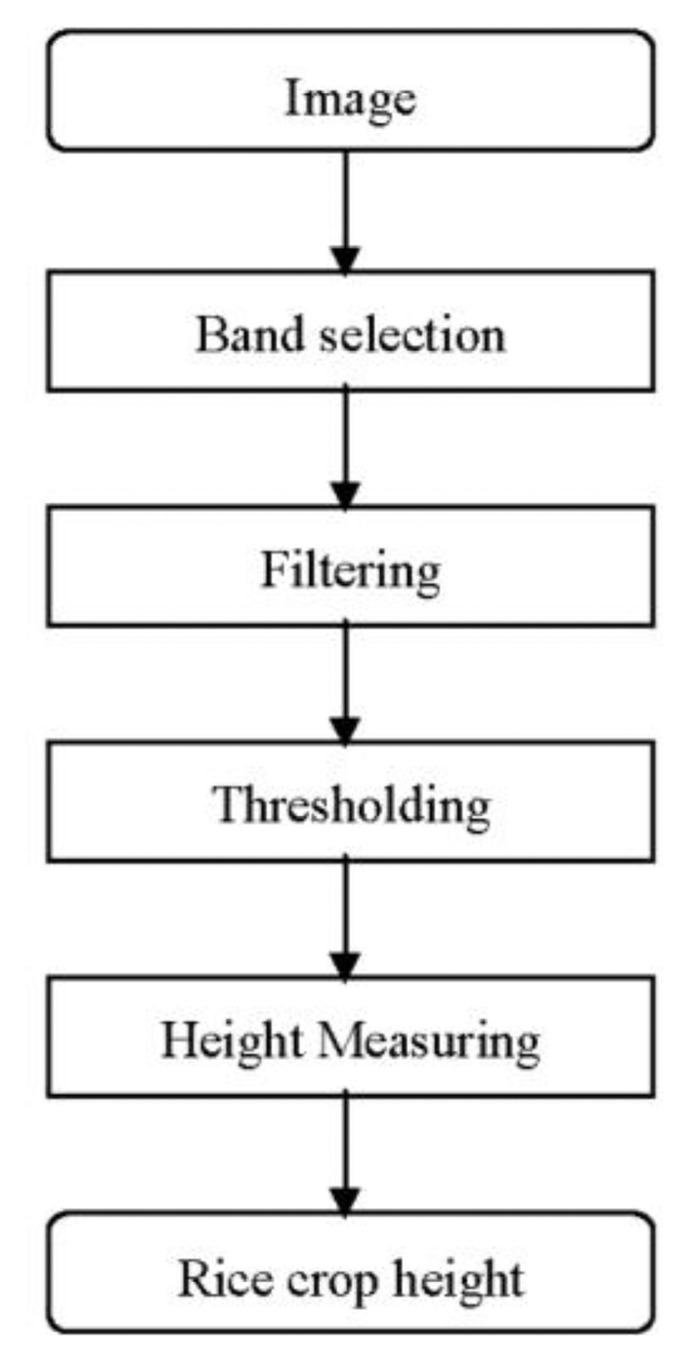
The rice crop height measurement flowchart.

The images acquired by the field server digital camera are composed of rice plants, the marker bar and the other areas such soil, cloud, and sky. The marker bar must be detected for indirect measurement of rice crop height. Band selection is used to remove redundant features. Filtering is used to extract the marker bar features. Thresholding is applied to separate objects and thus find the boundaries of the marker bar. The marker bar is detected and compared with the initial marker bar for measuring rice crop height. A flowchart of this method is shown in Figure 3 and the details are described in the following four subsections, which cover the topics of band selection, filtering, and image thresholding [38].

#### 2.3.1. Band Selection

Band selection [39] is the practice of choosing some subset of image data for further analysis. The data from many experiments often include redundant information thus band selection is used to discard information that is unnecessary for the problem at hand. By removing redundant features from the data, band selection can significantly improve the overall analysis performance.

**Figure 4.**
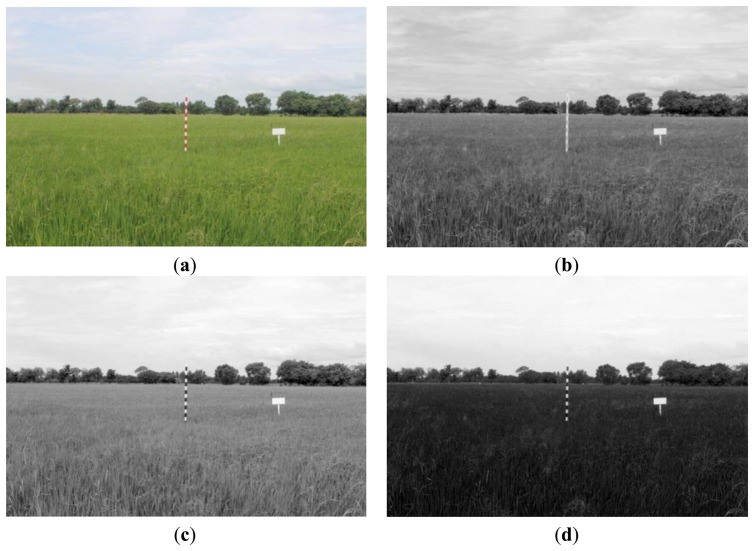
(**a**) RGB image; (**b**) Red band Image; (**c**) Green band Image; (**d**) Blue band Image.

Our digital camera provides images that use the RGB color model (Figure 4a). The color value of each pixel is composed of three components, namely red, green and blue. The marker bar is designed as alternating white and red colors. The alternating color pattern is easy for humans to detect and evaluate the height. In contrast, the alternating colors are harder to detect in digital image analysis than a single homogeneous color, since a homogeneous value for the whole object is easy to separate from other objects. We initially selected the red band to use as the primary feature in our analysis because in the red band, both the white and red colors of the marker bar will have high intensity values. In contrast, in either the green or the blue band, white areas will have high values but red areas will have low values.

Figure 4b for a red band shows that the whole marker bar has characteristics of a homogenous area and the marker bar values are uniformly high (white color). In contrast, both (c) and (d) in Figure 4 for green and blue bands show that the values of the marker bar alternate between high and low (white and black). Thus, the red band image is suitable to use for distinguishing between the marker bar and the rice crop.

Unfortunately, using only a single band of red, green or blue values in the analysis is not robust when the environment changes. Color indices [1,40] including excess green, excess red and excess blue have been shown to increase resistance to noise when the environment changes. The equations for calculating excess green, excess red and excess blue features are given as:

(1) ExcessRed=2R−G−B

(2) ExcessGreen=2G−R−B

(3) ExcessBlue=2B−G−R

where *R*, *G*, *B* are intensities of red, green and blue, respectively.

**Figure 5.**
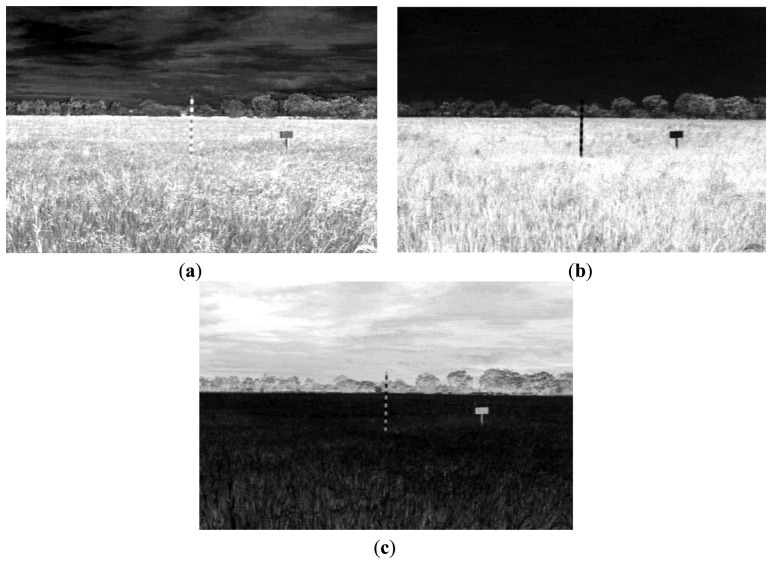
(**a**) Excess red image; (**b**) Excess green image; (**c**) Excess blue image.

Excess green was selected to be the primary feature in our analysis since, as shown in Figure 5, in excess green, values of both white and red colored areas of the marker bar are low and the marker looks homogeneous. This is not true for excess red and excess blue features. Eventually, excess green is most suitable to detect the marker bar.

In excess green image, the whole marker bar looks uniform and different from other objects. The observed marker bar can be estimated by choosing the longest vertical line. However, we found that the color of whole marker bar in the excess green image is not completely uniform so the white and red strips areas are still separated. As a result, the direct measurement of the crop height from the longest vertical line may be erroneous. Moreover, other environmental effects such as light wind, rain, *etc.*, may vary the intensity value in the excess green image. In order to measure the height of the rice crop efficiently under varied conditions, feature extraction step is needed. This feature extraction must transform the whole marker bar into a uniform object and be robust to various environmental factors.

#### 2.3.2. Filtering

Spatial filters are often used to extract features in image processing. The Laplacian filter [41,42] can be applied to extract edge features. The Laplacian of an image locates regions of large and sudden intensity change. The Laplacian *L*(*x*,*y*) of an image with pixel intensity values *I*(*x*,*y*) is given by:

(4) $$L(x,y)=\frac{\partial^{2}I}{\partial x^{2}}+\frac{\partial^{2}I}{\partial y^{2}}$$

**Figure 6.**
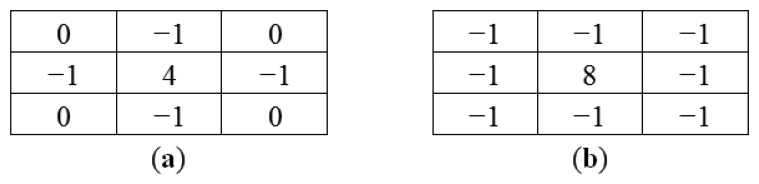
Laplacian filter. (**a**) 4-neighbor; (**b**) 8-neighbor.

**Figure 7.**
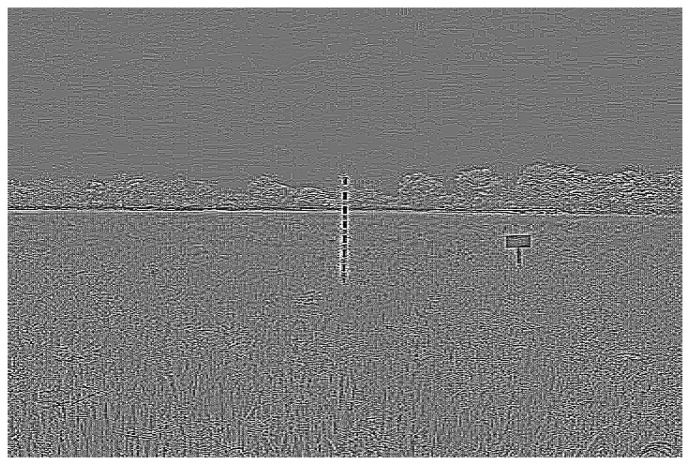
Filtered excess green band image with 4-neighbor Laplacian filter.

This equation can be computed using a convolution filter in the spatial domain. Since the input image is represented as a set of discrete pixels, a discrete convolution kernel can be approximated from the second derivatives in the definition of the Laplacian operation. Two commonly used small kernels with 3 × 3 pixels are shown in Figure 6. Figure 7 shows the red band image after application of this filter.

Result in Figure 7 shows that Laplacian filter can be used to detect the edges of the marker bar. However the edges of the marker bar show gaps, hence it is hard to separate the marker bar from other areas or objects.

**Figure 8.**
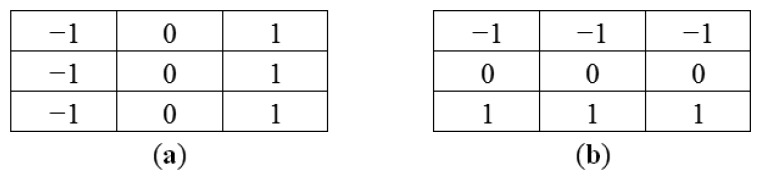
Directional filter. (**a**) *x*-direction; (**b**) *y*-direction.

Directional filters can be designed for any direction within a given space. For images, *x*- and *y*-directional filters are commonly used to compute derivatives in their respective orientations. Figure 8 shows kernel examples of a 3 × 3 pixels for x-direct and *y*-direction filters.

(5) $$F(x,y)=\frac{\partial I}{\partial x}$$

**Figure 9.**
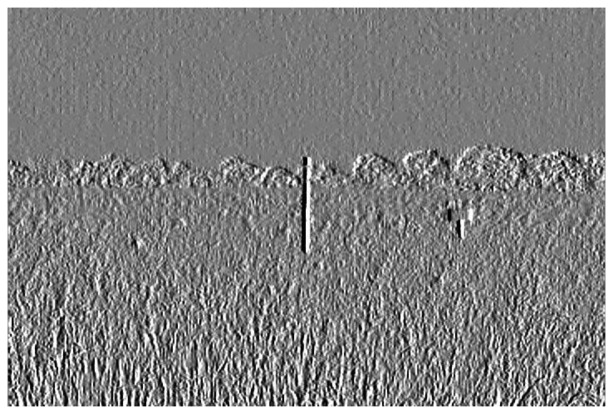
Filtered excess green image with *x*-direction filter.

**Figure 10.**
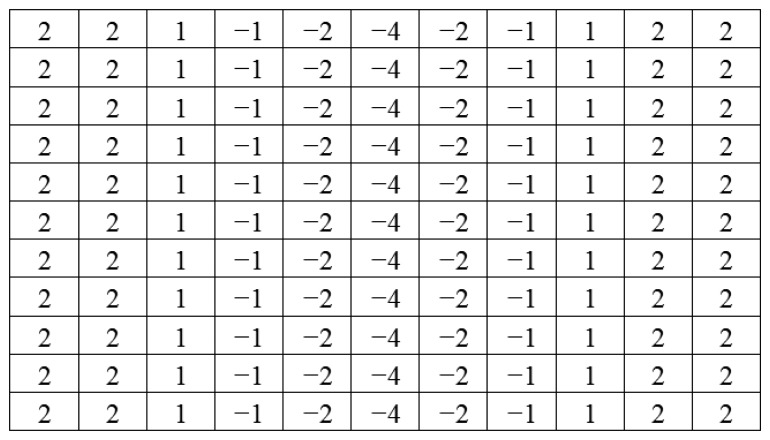
Modified filter.

**Figure 11.**
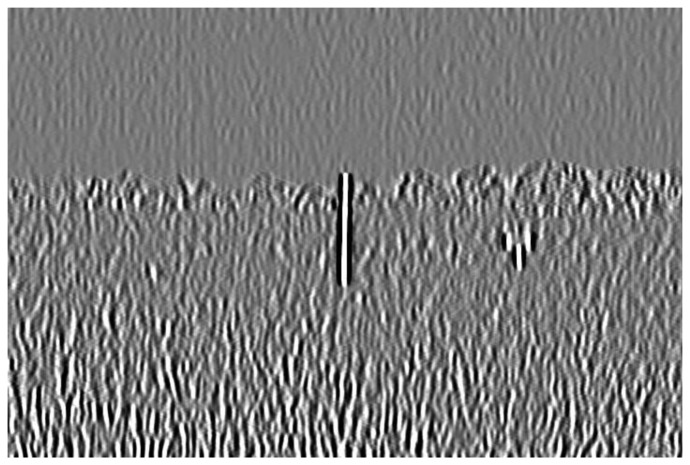
Filtered excess green image with modified filter.

Since the marker bar is vertical, we use a directional filter designed to find sudden gradients in the x direction, in order to extract the long edges parallel to the y-axis. Figure 9 show that after directional filtering, the filtered red band image has a clear long edge in the marker bar area. Though, there are still noise pixels such the edges of trees and rice stalks. Next, we modified the filter by integrating the Laplacian and directional filters. The modified filter is designed so that the kernel is assigned similarly to the Laplacian filter as second derivatives and also has the directional characteristic to find edges orthogonal to the width (*x*-axis). We deliberately assigned the values and set the size of the kernel to be appropriate for the size of the marker bar feature in the image. In this case, we observed that the width of marker bar in the image is about 5 pixels in the *x*-axis. Thus, we assigned the kernel size to be 11 pixels (about two times the width of the marker bar). Like the Laplacian filter, the five columns located in the center of the kernel in the *x*-axis were defined as negative-values since they was intended to similar to the marker bar that has low or negative value of excess-green, whereas the three columns located in the leftmost and the three columns located in the rightmost of the kernel in the *x*-axis were given as positive-values since the surrounding area of the mark bar has high intensity values in the excess-green color space. Also like directional filter in the *x*-axis, all the values in each column have the same values. When the filter slides to the marker bar, the multiplication of marker bar area (negative value multiply by negative value) and the other areas (positive value multiply by positive value) will produce high values. Figure 10 shows the 11 × 11 kernel for the modified filter. The excess green image after applying the modified filter is shown in Figure 11.

Figure 11 shows that modified filter clearly differentiates between the marker bar and other objects. In order to obtain the fittest discrimination between marker bar and the surrounding areas, the kernel filter must be optimized and it depends on the size of the marker bar in the image. The kernel is fairly sensitive to changea of resolution and distance between a camera and a marker bar. For the example; when a resolution of the image is lower or a marker bar is closer to a camera, the marker bar in image will be larger. As the result, the kernel will be defined as larger. In the other hand, if a resolution of the image is higher or a marker bar is further from a camera, the marker bar in the image will be constricted. Hence, the kernel should be assigned smaller.

#### 2.3.3. Thresholding

**Figure 12.**
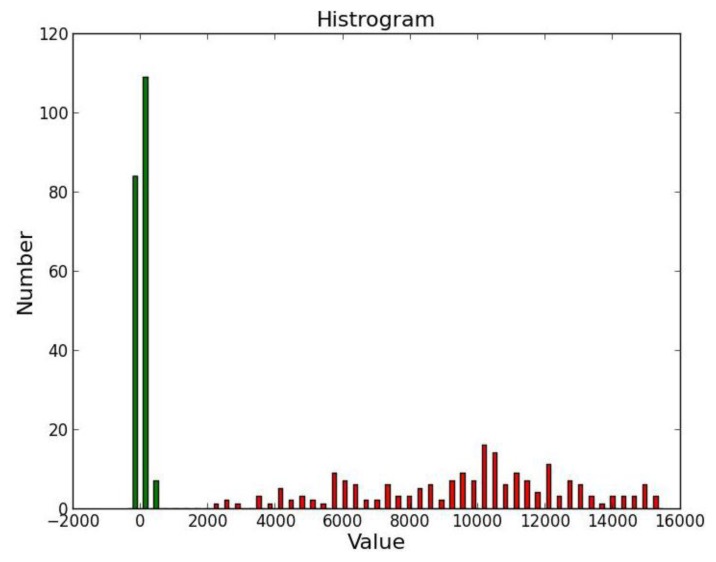
Histogram space between samples of marker bar area and other areas.

**Figure 13.**
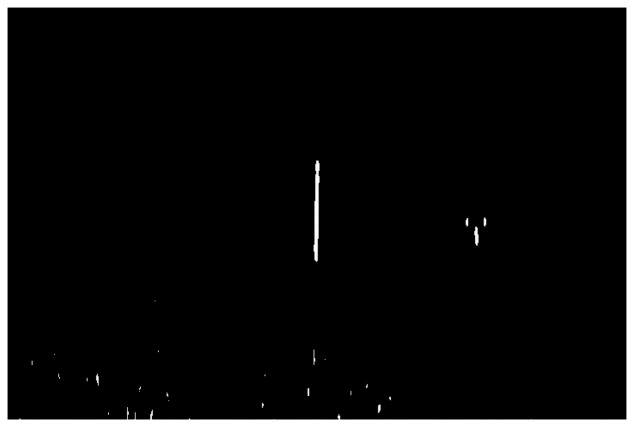
Resultant binary image.

Thresholding [43] is the simplest method of image segmentation. The objective of image segmentation is to simplify or change the representation of an image into something that is more meaningful and easier to analyze. We used thresholding to separate the image into pixels associated with the marker bar from others. Thresholding is applied to turn a gray-scale image into a binary image by applying a clip-level or threshold value. A gray-scale value above the threshold is set to one (white color) and a value equal to or below the threshold is set to zero (black color). The key to this method is to select an appropriate threshold value for the task. Here, we selected 200 samples of marker bar area and 200 samples of other areas such sky, tree and rice and put them into histogram-space (Figure 12). We deemed that they can be obviously separated into two groups; high value for marker bar area and low value for other areas. After that, we determined the appropriate value of threshold as 2,000 by varying values of threshold that provides the district discrimination between two separated groups (marker and non-marker). The resultant binary image (Figure 13) is separated into two types of pixels: (1) marker bar candidates with a pixel value of 1; (2) non-marker bar pixels with a value of 0:

(6) $$R(D(x,y))=\left\{ \begin{array}{ll} 1, & D(x,y)>\text{Threshold}\\ 0, & D(x,y)\leq \text{Threshold} \end{array}\right.$$

where *R*(*D*(*x*,*y*)) is one for masked marker bar, and zero for unmasked marker bar, and *D*(*x*,*y*) = values of image at pixel of (*x*,*y*).

#### 2.3.4. Height Measurement

**Figure 14.**
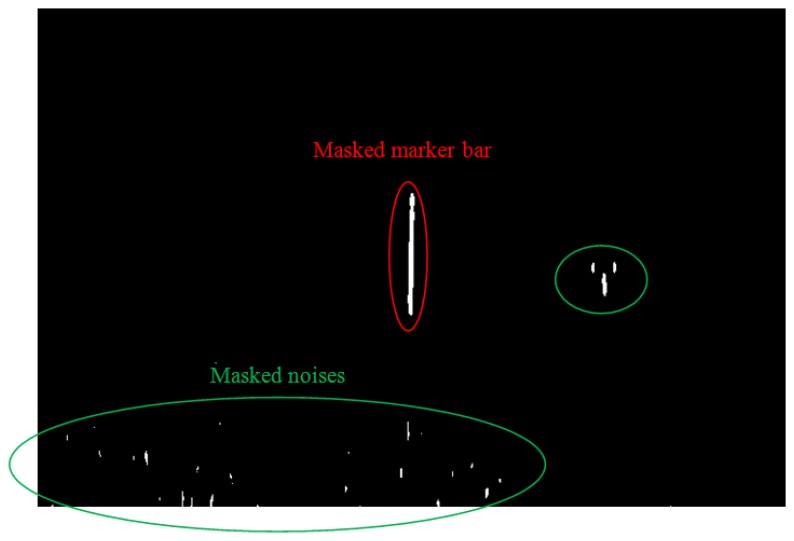
Resultant binary image; masked marker bar (red circle) and masked noises (green circle).

In the resultant binary image (Figure 14), there are a number of vertical white lines, including the marker bar plus other noise features. The marker bar is longer than any of the noise lines. Therefore, we identify and locate the longest vertical line in the image as the marker. We already know the height of the full marker bar (unobscured by rice). We can manually translate that into pixels. The rice crop height is the height of the hidden part of the marker bar which can be obtained by comparing the height of the detected marker bar with the height of the initial or full marker bar. Equation (3) shows the calculation for computing the rice crop height result. Our proposed method provides a representative height of the rice crop in this field:

(7) $$H=\frac{I-M}{I}L+R$$

where *H* is the height of rice crop in centimeters, *I* is the initial marker bar height in pixels, *L* is the initial marker bar height in centimeters, *M* is the detected marker bar height in pixels, and *R* is the initial height of rice crop from the first image.

**Figure 15.**
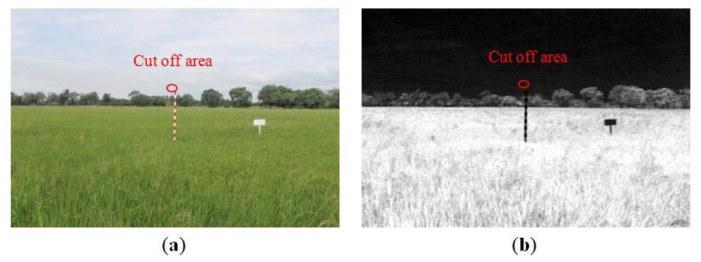
Cut off area (red circle) in (**a**) RGB and (**b**) modified filtered excess green images.

Marker bar and the background that are close to the marker bar should not have the same characteristics since it is hard to separate them. In this case, in the RGB image (Figure 15a), the white top marker bar resembles the sky. In the excess green feature band (Figure 15b), the value of topmost white stripe on the marker bar is also close to the value of the sky. The white top of the marker bar and the sky (white color) cannot be distinguished. The filtering cannot extract the edges and image segmentation cannot separate the objects. Therefore, initial marker bar height is essential to modify. We adjusted the height of our referenced region by excluding the white top of the marker bar that cannot be separated from the sky.

## 3. Experimental Results

The rice crop area images were taken at 10:30 AM every day from 1 July to 17 August 2012. Local Thai Meteorological Department staff recommended that images be taken in the morning as clouds and precipitation has less of an effect than at other times of the day. In total 48 images were used in this experiment. We observed the color of rice changed over time from green to gold colors. Furthermore, the rice field conditions also varied. Here, we divided the conditions into five categories, namely, (1) normal; (2) darkness or low light intensity; (3) brightness or high light intensity; (4) drizzle (which happens after the rain fall); (5) rainfall.

A value for initial rice crop height is needed for the height calculation. On 1 July 2012 the rice crop height was measured by visual inspection of the image and found to be 77.0 cm. This value was defined as the initial rice crop height. The rice crop in this field was harvested on 18 August 2012. Thus in the daily image series, there are 48 images to evaluate the proposed method.

To provide a referenced height so that we can evaluate the accuracy of our measures, we used manual estimation. The marker bar was designed for human estimation, and the rice crop height can be reliably evaluated by a human observing an image. To validate the referenced results obtained by manual estimation, we surveyed the rice crop field and directly measured rice crop height in this field using tap meters on the dates of 14 July and 12 August 2012. The results based on human estimation and surveyed data are very close, 98.24% accuracy on 14 July 2012 and 98.80% accuracy on 12 August 2012. Thereby the results of human evaluation appear to be reliable enough to use as a standard in reviewing the results of our proposed method.

We use relative error index given as:

(8) $$\text{Relative error}(\%)=\frac{\left|\text{Estimated Result}-\text{Referenced Result}\right|}{\text{Referenced Result}}\times 100$$

to express the accuracy of our experimental results. For performance comparison, we applied our method for automatic rice crop height estimation using both the excess green and also the red band color features. Our method is then applied to full 48 daily images and the results from our proposed method with red band color and excess green features are given in Tables 1 and 2, respectively.

The averaged relative error of proposed method with the red band color feature is 24.85% with the standard deviation of 43.45% while the averaged relative error of proposed method with the excess green feature is about 3.34% with the standard deviation of 5.23%. These results show that proposed method can accurately measure rice crop height using the excess green feature, while the red band does not provide satisfactory accuracy. The *t*-test [44] is used to statistically compare the performances of our method with red band color and excess green features, and the *t*-statistics is equal to 5.529 with the *p*-value of 1.109 × 10^−6^. Hence, we can conclude that, for our proposed method, the excess green color feature provides significantly better performance than the red band color feature.

The results in Table 1 indicate that while the red band feature can measure some images accurately, but other images with low light intensity caused by clouds, rainfall or drizzle happening after the rainfall produce high degrees of error. Moreover, after the color of rice changes from green to golden, the red band does not provide accurate measurements. As shown in Table 2, the excess green feature provides good accuracy for all images, except one image taken in the rain.

**Figure 16.**
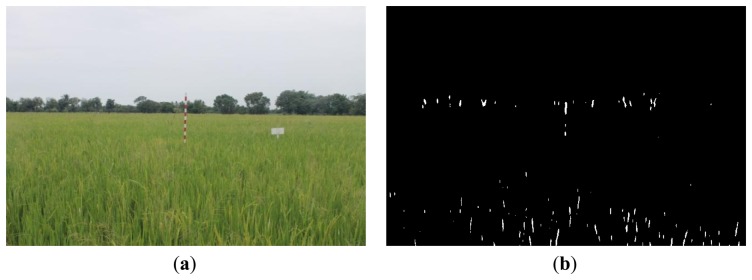
Comparison between (**a**) RGB and (**b**) resultant binary images using proposed method with red band feature on 18 July 2012.

**Figure 17.**
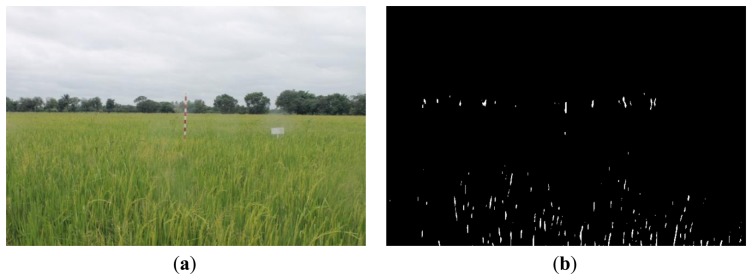
Comparison between (**a**) RGB and (**b**) resultant binary images using proposed method with red band feature on 19 July 2012.

**Figure 18.**
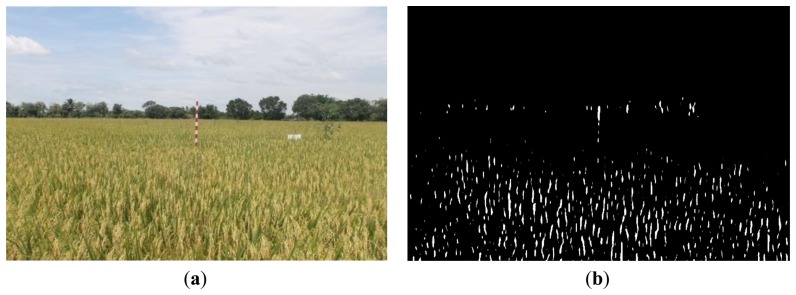
Comparison between (**a**) RGB and (**b**) resultant binary images using proposed method with red band feature on 12 August 2012.

**Figure 19.**
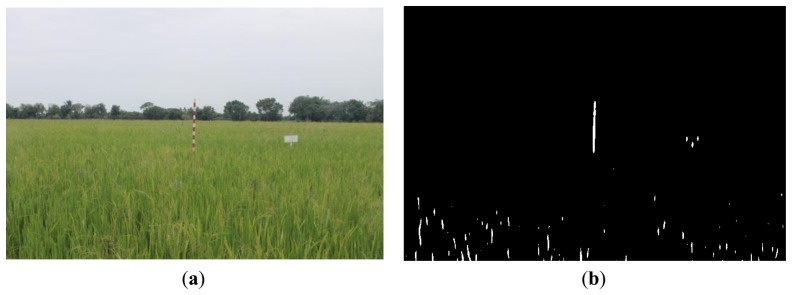
Comparison between (**a**) RGB and (**b**) resultant binary images using proposed method with red band feature on 18 July 2012.

**Figure 20.**
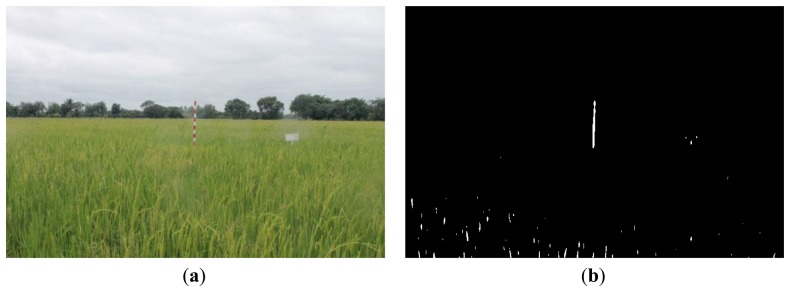
Comparison between (**a**) RGB and (**b**) resultant binary images using proposed method with excess green feature on 19 July 2012.

**Figure 21.**
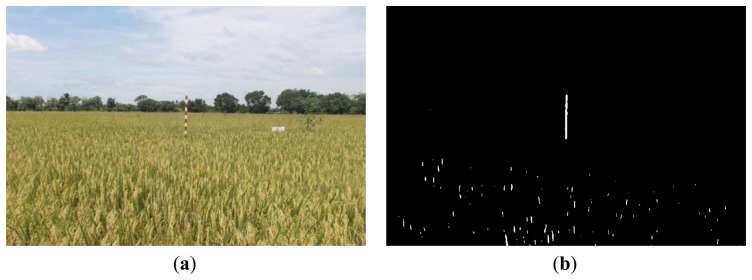
Comparison between (**a**) RGB and (**b**) resultant binary images using proposed method with excess green feature on 12 August 2012.

Figures 16, 17 and 18 show the red band resultant binary images for several cases in Table 1 where the calculated height showed poor accuracy. Figures 19, 20 and 21 show the binary images based on the same input data, using the excess green feature, illustrating that this feature provides far better results.

Figures 16, 17 and 18 show that the marker bar areas in the RGB image (assessed by the human judgment) and located by our method in the binary result images are not similar. Figure 16 shows field conditions with darkness or low light intensity. Figure 16 is disturbed by drizzle occurring after the rainfall, and in Figure 17 the rice has matured and turned golden in color. These examples indicate that red band feature is rather sensitive to noise. In contrast, Figures 19, 20 and 21 show good correspondence between the marker bar area in the resultant binary image and that in the RGB image except for the drizzle and the changing rice color. Thus the excess green feature is more robust against changes in the crop and weather conditions.

For variation of light intensity, light intensity can be observed by the pyranometer on the field survey device and the light intensity was divided into three levels: low light intensity (less than 400 W/sq.m., darkness), middle light intensity (between 400 and 1,000 W/sq.m., normal) or high light intensity (more than 1,000 W/sq.m., brightness). The low light intensity gives less contrast. Hence the result of segmentation using the red band feature may be inaccurate. On the other hand, the high intensity provides the contrast of the image that can help to easily separate objects. Fortunately, the proposed method with excess green can overcome this problem effectively. In cases of both low and high light intensity, three components (R, G, B) have all lower or higher values, respectively. It does not take effect to excess green equation.

As for changes on the rice, when rice has matured, its color will change from green to gold color. In the red band image, the intensity of rice will be higher when rice changes from green to gold color. As the result, the marker bar and rice have more similarity. It is hard to distinguish them and the results are likely to be incorrect. Again, the proposed method with excess green efficaciously manages the problem. Although the intensity of the red band increases, the distinction between excess green values of the marker bar and rice crop are sufficiently high, so it can separate the marker bar from the rice crop.

As for the drizzle occurring after a rainfall, it can cause some area images to become blurry. However, it has few effects. Since the red band image is sensitive to noise, it will take effect and affect the segmentation step to separate incorrectly. However, it does not affect our proposed method using excess green feature.

**Figure 22.**
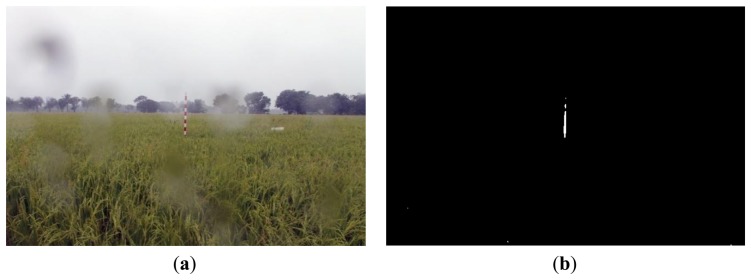
Comparison between (**a**) RGB and (**b**) resultant binary images using proposed method with excess green feature in case of rainfall on 25 July 2012.

For the rainfall, if rain is falling when the image is acquired, this seriously reduces the accuracy of the result for both the red band and the excess green feature since the rainfall reduces the image quality (blurred image). Figure 22 shows that RGB image was taken while it was raining on 25 July 2012 and the resultant binary image using the excess green feature was not able to correctly segment the marker bar. The masked marker bar in the resultant binary image looks very small (about half size) when comparing with the marker bar in RGB image. When the detected marker bar is computed in Equation (7), the result of rice crop height will be very high (about one and half times). Also the result of the rice crop height is hugely inaccurate, with a relative error value of 36.41%. In addition, the blurred image affected by rain perhaps could be calculated in the spatial frequency domain for easily distinguishing and improving the resultant accuracy.

**Figure 23.**
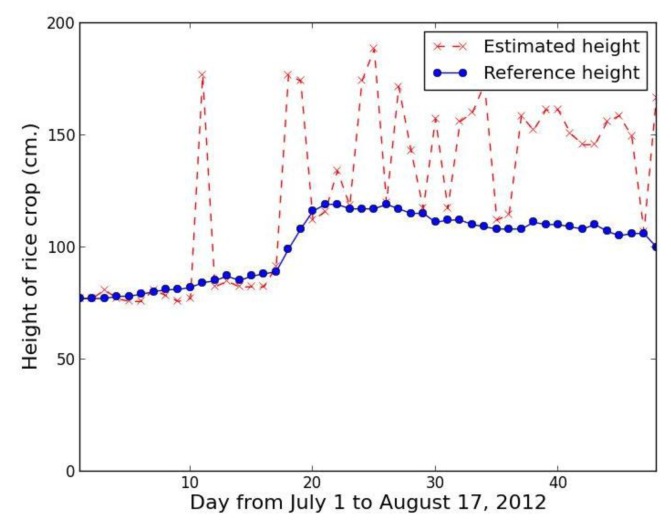
Rice crop height series from 1 July to 17 August 2012 using proposed method with red band feature.

**Figure 24.**
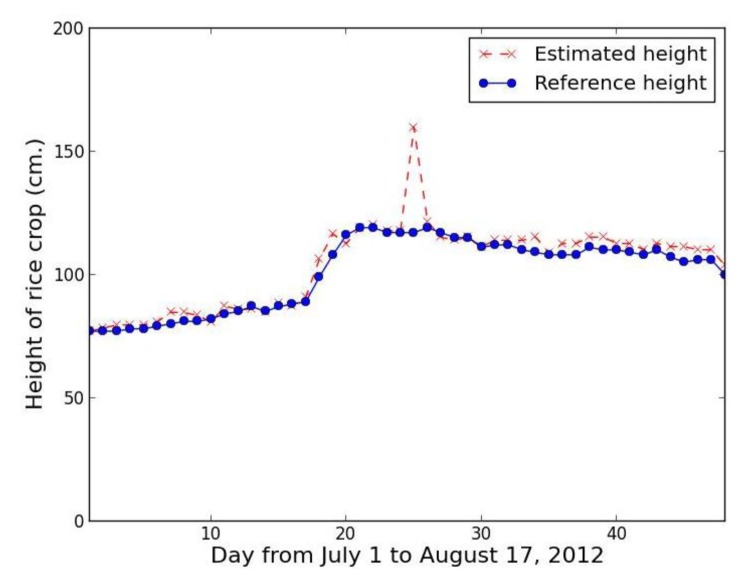
Rice crop height series from 1 July to 17 August 2012 using proposed method with excess green feature.

Collecting rice crop height data every day makes it possible to analyze the data in time series for rice growth state monitoring, yield estimation, and classification of the type of rice crop. Figure 23 shows a time series based on our proposed method using the red band feature, while Figure 24 shows a comparable series using the excess green feature.

The time series results confirm that proposed method using the red band feature is not smooth, with lots of error, while the proposed method using the excess green feature closely tracks the referenced height with the exception of one point, which is the rainfall case of 25 July 2012.

Moreover, we could possibly introduce a hybrid data analysis method since the other sensors of the field sever provide other information to support estimation of rice crop height. The rainfall is possibly one factor that affects the accuracy in the crop height measurement. The measurement may be inaccurate or unreliable when taken during the rainfall. To avoid directly using the record affected by rain, the reliable neighboring records that are not affected by rain are used to assist the estimation of the height by an interpolation method. For example, at 10.30 A.M. on 25 July 2012, the rain-gauge measures a rain rate of 10.2 mm/h. This implies that it was raining around the studied area.

**Figure 25.**
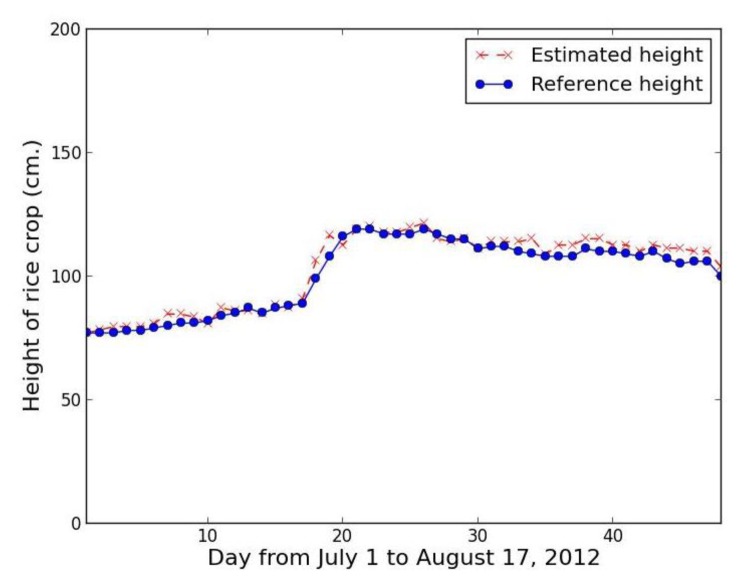
Rice crop height series from 1 July to 17 August 2012 using the hybrid data analysis method with excess green feature.

Consequently, our system used the value calculated by neighboring interpolation method instead of the record of 25 July 2012 by 117.7 cm. The resultant error is reduced from 36.43% to 0.58%. Light intensity is a major variable to directly affect an image quality. In the case of using red band, the low light intensity gives an error. For the same reason, the result using proposed method should be replaced by the result using the neighboring interpolation method. The hybrid data analysis method with excess green feature improves the estimated results efficaciously (Figure 25). The average relative error is only 2.63%.

## 4. Discussion

By based on our experimental results, we can conclude that the proposed method performs better with the excess green than with the red band for the red-white stripe marker bars since the excess green can capture the differences between the marker bar and the surrounding environment. However, if a different marker bar is used, different features may be needed. We chose to use a red-white stripe marker bar since they are commonly employed in agricultural fields. An alternative color pattern allows the height of a crop to be easily evaluated by a farmer by counting the vegetation-covered blocks on the bar. From the image analysis perspective, the uniform color bar should be easier to detect than using the stripe bar. Nevertheless, the alternative color pattern was employed for easy comparison between the proposed method and manual evaluation.

**Figure 26.**
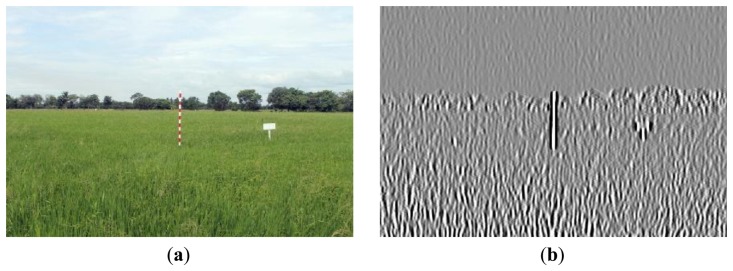
Comparison between (**a**) RGB and (**b**) 7 × 7 modified filtered excess green images with resolution of 432 × 288 on 1 July 2012.

**Figure 27.**
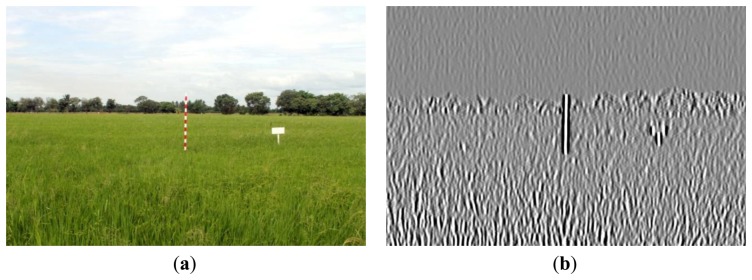
Comparison between (**a**) RGB and (**b**) 15 × 15 modified filtered excess green images with resolution of 1,008 × 672 on 1 July 2012.

**Figure 28.**
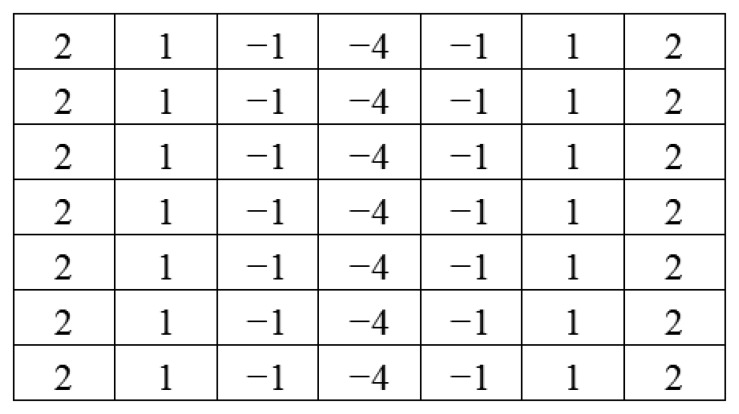
Modified filter with size of 7 × 7.

**Figure 29.**
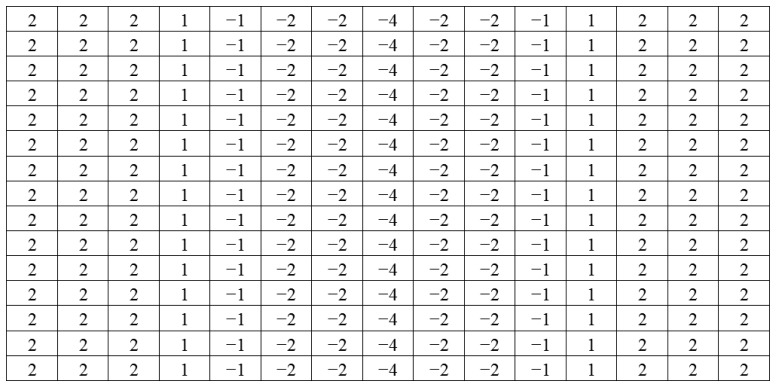
Modified filter with size of 15 × 15.

In our experiment, the distance between the marker bar and the field sever was fixed throughout the experiment. Hence, if the similar experiment is conducted by placing the field sever and marker bar at different distances, the parameter used in the crop height measurement may need to be adjusted. In fact, if a marker bar is placed further away from the camera, the width of marker bar appeared on the captured images will be smaller, and if a mark bar is placed closer to the camera, the width will be larger. In the experiment, the width of the marker bar was 5 pixels, and we chose the optimized filter of size 11 × 11 pixels or equal to twice the size of the marker bar plus 1. To test varying size of marker bar in the image, for instance, the original RGB image acquired from filed server on 1 July 2012 was resampled from 720 × 480 pixels to 432 × 288 pixels, and the width of the marker bar is reduced to 3 pixels (Figure 26a). Similarly, we also resampled the original image to a size of 1,008 × 672 pixels to enlarge the width of the marker bar to 7 pixels (Figure 27a). In the first case (marker bar width of 3 pixels), the suitable filter have a size of 7 × 7 (Figure 28) whereas for the enlarged image (marker bar width of 7 pixels), the appropriate size of the filer is 15 × 15 (Figure 29). The filtered images for both cases are displayed in Figures 26b and 27b for the marker bar width of 3 and 7 pixels, respectively. For both cases, the marker bar is clearly separate from the surrounding areas. However, if a marker bar is too far away from a camera (e.g., the width is less than one pixel), our proposed method may fail to capture a marker bar since it will be very difficult to extract such a thin object from an image. Nevertheless, in actual implementations, it is unlikely one would encounter this extreme situation. In contrast to the pervious situation, if a marker bar and a field server are too close to each other, the nonlinearity effect of camera may severely distort the captured image in such a way that the length of captured marker bar may be significantly deviated from the actual length. As a result, the field server and marker bars should be not too far or too close to each other.

In some scenarios, we may want to measure the height of rice at multiple points in the field to obtain more accuracy the inference of the growth stage. In these scenarios, our proposed method should be adapted to estimate the length of multiple marker bars. To achieve this goal, multiple filters with different sizes should be used to extract marker bar at various distances from the field survey. Furthermore, instead of selecting the longest vertical line as the marker bar candidate, our proposed method should select the first *n* longest marker bars if *n* marker bars are placed in the field.

## 5. Conclusions

This paper presents an automatic method for rice crop height measurement using a field server and image processing techniques. A field server equipped with a digital camera provides the rice field images including rice stalks and a calibrated marker bar. In the height analysis based on digital image processing, band selection helps to reduce unnecessary features. Filtering enhances features relevant to the problem. Thresholding is used to separate the marker bar from other parts in the captured images. The detected marker bar is compared with the initial marker bar to provide the inference of the rice crop height in the field. However, the representative height of the rice crop is just a sample point in the crop field, the resultant height of rice crop is roughly obtained. If the extensive accuracy of the crop field is required, multiple marker bars can be comprehensively installed in the crop field to measure the heights in multiple areas. Besides, if camera and marker bar are at fixed locations, a region of interest (ROI) in the image, a selected subset of samples within a dataset defined for a specific purpose, can be defined to improve accuracy and reduce computing time since unnecessary features such as sky, and trees are removed in computation. On the other hand, if the camera and marker bar are at flexible locations, it is essential to assign the kernel of the optimized filter and initial parameters is observed and defined such as initial marker bar both in reality and imagery is also cut off the area when background is changed.

The advantages of the method are a reduction in cost and an increase in speed by providing a real time monitoring system. Since the proposed method employed a simple image processing algorithm, this method can be implemented in an embedded system installed directly at the field server. The device can calculate the resultant height without transferring actual images to a remote server. Furthermore, our proposed algorithm to measure a plant's height can apply for other plants that have similar characteristics as rice such as wheat since they are hard to directly measure but it can be indirectly measured.

## Figures and Tables

**Table 1.** Comparison between estimated results using red band feature and referenced results.

**Date of Image**	**Color of** **Rice**	**Condition of** **Field**	**Referenced** **Result (cm)**	**Estimated** **Result (cm)**	**Relative** **Error (%)**
1 July 2012	Green	Normal	77.0	77.0	0.00
2 July 2012	Green	Normal	77.0	77.0	0.00
3 July 2012	Green	Normal	77.0	80.9	5.04
4 July 2012	Green	Normal	78.0	77.0	1.28
5 July 2012	Green	Normal	78.0	75.7	2.94
6 July 2012	Green	Normal	79.0	75.7	4.17
7 July 2012	Green	Normal	80.0	80.9	1.10
8 July 2012	Green	Normal	81.0	78.3	3.34
9 July 2012	Green	Normal	81.0	75.7	6.54
10 July 2012	Green	Normal	82.0	77.0	6.10
11 July 2012	Green	Darkness	84.0	176.6	110.29
12 July 2012	Green	Normal	85.0	82.2	3.32
13 July 2012	Green	Normal	87.0	84.7	2.57
14 July 2012	Green	Normal	85.0	82.2	3.32
15 July 2012	Green	Normal	87.0	82.2	5.54
16 July 2012	Green	Normal	88.0	82.2	6.62
17 July 2012	Green	Normal	89.0	91.2	2.51
18 July 2012	Green	Darkness	99.0	176.6	78.43
19 July 2012	Green	Drizzle	108.0	174.0	61.17
20 July 2012	Golden	Normal	116.0	111.9	3.50
21 July 2012	Golden	Normal	119.0	115.8	2.67
22 July 2012	Golden	Normal	119.0	133.9	12.56
23 July 2012	Golden	Normal	117.0	118.4	1.21
24 July 2012	Golden	Darkness	117.0	174.1	48.77
25 July 2012	Golden	Rainfall	117.0	188.3	60.94
26 July 2012	Golden	Normal	119.0	119.7	0.59
27 July 2012	Golden	Darkness	117.0	171.5	46.56
28 July 2012	Golden	Normal	115.0	143.0	24.35
29 July 2012	Golden	Normal	115.0	117.1	1.84
30 July 2012	Golden	Darkness	111.0	157.2	41.65
31 July 2012	Golden	Normal	112.0	117.1	4.57
1 August 2012	Golden	Normal	112.0	155.9	39.23
2 August 2012	Golden	Darkness	110.0	159.8	45.29
3 August 2012	Golden	Normal	109.0	172.8	58.50
4 August 2012	Golden	Brightness	108.0	111.9	3.65
5 August 2012	Golden	Brightness	108.0	114.5	6.05
6 August 2012	Golden	Darkness	108.0	158.5	46.79
7 August 2012	Golden	Normal	111.0	152.1	36.99
8 August 2012	Golden	Darkness	110.0	161.1	46.47
9 August 2012	Golden	Darkness	110.0	161.1	46.47
10 August 2012	Golden	Normal	109.0	150.8	38.32
11 August 2012	Golden	Normal	108.0	145.6	34.80
12 August 2012	Golden	Normal	110.0	145.6	32.35
13 August 2012	Golden	Normal	107.0	155.9	45.74
14 August 2012	Golden	Normal	105.0	158.5	50.98
15 August 2012	Golden	Normal	106.0	149.5	41.00
16 August 2012	Golden	Brightness	106.0	106.7	0.72
17 August 2012	Golden	Normal	100.0	166.3	66.29

**Table 2.** Comparison between estimated results using excess green feature and referenced results.

**Date of Image**	**Color of** **Rice**	**Condition of** **Field**	**Referenced** **Result (cm)**	**Estimated** **Result (cm)**	**Relative** **Error (%)**
1 July 2012	Green	Normal	77.0	77.0	0.00
2 July 2012	Green	Normal	77.0	78.3	1.65
3 July 2012	Green	Normal	77.0	79.5	3.30
4 July 2012	Green	Normal	78.0	79.5	1.98
5 July 2012	Green	Normal	78.0	79.5	1.98
6 July 2012	Green	Normal	79.0	80.81	2.30
7 July 2012	Green	Normal	80.0	84.6	5.78
8 July 2012	Green	Normal	81.0	84.6	4.48
9 July 2012	Green	Normal	81.0	83.4	2.91
10 July 2012	Green	Normal	82.0	80.8	1.45
11 July 2012	Green	Darkness	84.0	87.2	3.77
12 July 2012	Green	Normal	85.0	85.9	1.06
13 July 2012	Green	Normal	87.0	85.9	1.27
14 July 2012	Green	Normal	85.0	84.6	0.44
15 July 2012	Green	Normal	87.0	88.4	1.66
16 July 2012	Green	Normal	88.0	87.2	0.94
17 July 2012	Green	Normal	89.0	91.0	2.23
18 July 2012	Green	Darkness	99.0	106.2	7.31
19 July 2012	Green	Drizzle	108.0	116.4	7.78
20 July 2012	Golden	Normal	116.0	112.6	2.94
21 July 2012	Golden	Normal	119.0	118.9	0.04
22 July 2012	Golden	Normal	119.0	120.2	1.03
23 July 2012	Golden	Normal	117.0	117.7	0.58
24 July 2012	Golden	Darkness	117.0	117.7	0.58
25 July 2012	Golden	Rainfall	117.0	159.6	36.43
26 July 2012	Golden	Normal	119.0	121.5	2.09
27 July 2012	Golden	Darkness	117.0	115.1	1.59
28 July 2012	Golden	Normal	115.0	113.9	0.99
29 July 2012	Golden	Normal	115.0	115.1	0.12
30 July 2012	Golden	Darkness	111.0	111.3	0.29
31 July 2012	Golden	Normal	112.0	113.9	1.66
1 August 2012	Golden	Normal	112.0	113.9	1.66
2 August 2012	Golden	Darkness	110.0	113.9	3.51
3 August 2012	Golden	Normal	109.0	115.1	5.63
4 August 2012	Golden	Brightness	108.0	108.8	0.72
5 August 2012	Golden	Brightness	108.0	112.6	4.25
6 August 2012	Golden	Darkness	108.0	112.6	4.25
7 August 2012	Golden	Normal	111.0	115.1	3.73
8 August 2012	Golden	Darkness	110.0	115.1	4.67
9 August 2012	Golden	Darkness	110.0	112.6	2.36
10 August 2012	Golden	Normal	109.0	112.6	3.30
11 August 2012	Golden	Normal	108.0	110.1	1.90
12 August 2012	Golden	Normal	110.0	112.6	2.36
13 August 2012	Golden	Normal	107.0	111.3	4.04
14 August 2012	Golden	Normal	105.0	111.3	6.02
15 August 2012	Golden	Normal	106.0	110.1	3.82
16 August 2012	Golden	Brightness	106.0	110.1	3.82
17 August 2012	Golden	Normal	100.0	103.7	3.69
